# Computational identification and validation of alternative splicing in ZSF1 rat RNA-seq data, a preclinical model for type 2 diabetic nephropathy

**DOI:** 10.1038/s41598-018-26035-x

**Published:** 2018-05-16

**Authors:** Chi Zhang, Ken Dower, Baohong Zhang, Robert V. Martinez, Lih-Ling Lin, Shanrong Zhao

**Affiliations:** 1 Precision Medicine, Early Clinical Development, Pfizer Worldwide Research and Development, Cambridge, MA 02139 USA; 2 Inflammation & Immunology Research Unit, Pfizer Worldwide Research and Development, Cambridge, MA 02139 USA

## Abstract

Obese ZSF1 rats exhibit spontaneous time-dependent diabetic nephropathy and are considered to be a highly relevant animal model of progressive human diabetic kidney disease. We previously identified gene expression changes between disease and control animals across six time points from 12 to 41 weeks. In this study, the same data were analysed at the isoform and exon levels to reveal additional disease mechanisms that may be governed by alternative splicing. Our analyses identified alternative splicing patterns in genes that may be implicated in disease pathogenesis (such as *Shc1*, *Serpinc1*, *Epb4.1l5*, and *Il-33*), which would have been overlooked in standard gene-level analysis. The alternatively spliced genes were enriched in pathways related to cell adhesion, cell–cell interactions/junctions, and cytoskeleton signalling, whereas the differentially expressed genes were enriched in pathways related to immune response, G protein-coupled receptor, and cAMP signalling. Our findings indicate that additional mechanistic insights can be gained from exon- and isoform-level data analyses over standard gene-level analysis. Considering alternative splicing is poorly conserved between rodents and humans, it is noted that this work is not translational, but the point holds true that additional insights can be gained from alternative splicing analysis of RNA-seq data.

## Introduction

Diabetic nephropathy (DN) accounts for about 44% of patients starting treatment for kidney failure each year, and is the leading cause for end-stage renal disease that requires dialysis or renal replacement therapy for patient survival^1^. The underlying mechanisms of the disease progression are not fully understood. Although DN is generally considered to be a nonimmune-mediated degenerative disease, complement proteins and immunoglobulins can be detected in diseased glomeruli^2^.

Several groups have demonstrated that the obese ZSF1 rat is one of relatively few rodent models that mimic the disease progression of human type 2 DN^3,4^. Previously, we performed a high-resolution longitudinal study of age-matched lean and obese ZSF1 rats spanning from early DN to end-stage renal disease^3^. The analyses included metabolic endpoints, renal histology and ultrastructure, fibrosis biomarker evaluation, and gene-level RNA-seq analyses of glomerular-enriched tissue over the course of disease progression^3^.

RNA-seq is routinely used to profile transcriptome changes in disease versus normal tissues^5^. While most RNA-seq studies focus on expression at the gene level, it is now appreciated that the majority of mammalian genes can be alternatively spliced to generate multiple different isoforms that encode proteins with diverse, and even opposing, biological functions^6^. In the human Gencode annotation Release 25, the number of annotated transcripts is more than three times the number of annotated genes; for protein coding genes, this ratio increases to 7:1. It has also been reported that 50–60% of disease-causing mutations in the human gene mutation database affect splicing^7,8^. These findings have resulted in a growing interest in understanding alternative splicing patterns in disease. Moreover, targeting splicing regulatory proteins and/or specific key altered splicing events has emerged as a therapeutic strategy, for example, in rare diseases^9^ and cancers^10^.

Several computational tools have been developed to analyse alternative spliced transcripts in RNA-seq data^11^. These tools can be divided into two categories based on their functionality. Tools in the first category include Cufflinks^12^, RSEM^13^, Kallisto^14^, and Salmon^15^, which can be applied to analyse known or annotated transcript isoforms. In this study, we chose Salmon, one of the top performers in speed and accuracy based on our internal evaluation^16^, for this type of analysis. Tools in the second category include MISO^17^, MAJIQ^18^, and rMATS^19^, which can be used to analyse RNA-seq data at the exon level and to detect known and novel splicing events. These latter methods are especially useful for organisms with limited annotation on isoforms. We chose rMATS for this type of analysis in the present study.

The longitudinal RNA-seq data of isolated glomeruli from lean (non-diseased) and obese (diseased) ZSF1 rats were comprehensively analysed at both the isoform and the exon levels. We report several alternative splicing patterns in obese versus lean animals in genes involved in metabolism, immune response, podocyte dedifferentiation, and apoptosis. Our findings provide an example of how isoform and exon analyses can identify molecular changes that may contribute to disease pathogenesis, which would be overlooked in standard gene-level analyses. In addition, we validated by qRT-PCR the existence of selected novel mRNA isoforms that were identified computationally.

## Results

### RNA-seq analyses of ZSF1 glomeruli at the isoform level

The RNA-seq dataset was described in detail in a previous report^3^. Briefly, glomeruli were enriched from the kidneys of ZSF1 lean and obese animals at 12, 20, 24, 29, 34, and 41 weeks of age for RNA-seq library preparation. The study consisted of five lean and five obese RNA samples per age group with the exception of week 41, because one lean and two obese animals died over the study period. The metadata for all 57 RNA samples are listed in Supplementary Table S1.

The RNA-seq dataset was reanalysed using Salmon^15^ 0.8.2 and the Ensembl v87 rat transcriptome annotation. At each time point, the group average of transcript-level log_2_TPM (log2 of Transcript Per Million) values was calculated for lean and obese animals. The scatter plots of lean versus obese groups from this analysis are shown in Supplementary Fig. S1. Transcripts with over 16-fold TPM differences in each comparison were identified and labelled. Next, differential expression analyses between lean and obese animals at each time point were performed at both the gene and transcript levels using the Bioconductor package edgeR^20^. The numbers of differentially expressed genes and differentially expressed transcripts from this analysis are summarized in Table 1. In concordance with the gene-level analyses^3^, the number of differentially expressed transcripts increased with disease progression from weeks 12 to 41, and was particularly evident at weeks 34 and 41. The differentially expressed transcripts were then grouped by their corresponding genes to obtain the number of genes with one or more differentially expressed transcripts. At every time point, the number of genes with one or more differentially expressed transcripts was always higher than the corresponding number of differentially expressed genes. Thus, more differentially expressed genes were discovered at the isoform level than at the gene level.

**Table 1:** Numbers of differentially expressed genes (DE_gene) and differentially expressed transcripts (DE_tx) between obese and lean animals at each time point.

Week	DE_gene	DE_tx_gene	DE_tx
12	282	320	340
20	227	243	256
24	299	332	360
29	395	462	499
34	1152	1247	1389
41	1524	1635	1855

DE_tx_gene indicates the number of genes that correspond to the transcripts in DE_tx.

We were particularly interested in genes that displayed significant changes at the transcript level but not at the gene level, because such changes would be overlooked in gene-level analysis. To identify such genes, we proceeded as follows. For each gene, the FDR (false discovery rate) at the gene level (FDR_gene_) and the minimum FDR across all transcripts (min_FDR_trans_) were first calculated at all time points. Then, (−log_10_min_FDR_trans_) − (−log_10_FDR_gene_) was calculated at each time point and the maximum value for each gene across all time points was used to rank all the genes. The top 200 genes were visually inspected, and three DN-associated genes, *Shc1*, *Serpinc1*, and *Epb4.1l5*, were examined in detail.

**Figure 1:**
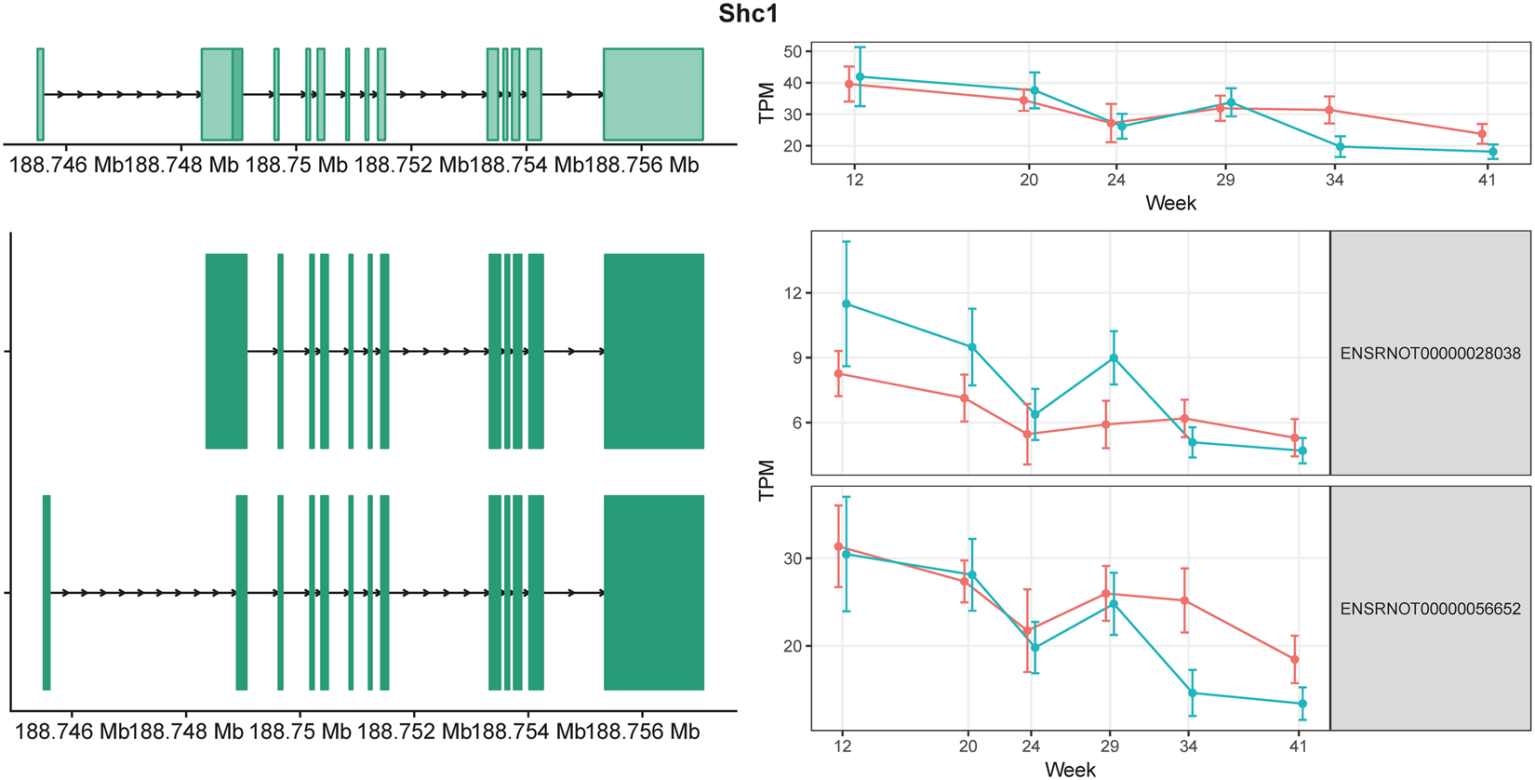
Exon structures and expression levels of *Shc1* and its two transcripts. The gene structure (top left) and isoform structures (bottom left) are shown with the chromosome coordinates on the left. *Shc1* is on the “+” strand. The exons are shown in green. The corresponding longitudinal TPM values estimated from the RNA-seq data are shown on the right. Lean and obese animals are indicated in red and blue, respectively. The error bars indicate the 95% confidence intervals.

*Shc1* (SHC adaptor protein 1) has two annotated isoforms in rat Ensembl v87: ENSRNOT00000028038 and ENSRNOT00000056652 because of alternative promoter usage (Fig. 1). ENSRNOT00000028038 can be translated to the p66^Shc^ protein isoform, which is predominantly found in epithelial cells^21^. ENSRNOT00000056652 can be translated to p52^Shc^ and p46^Shc^ protein isoforms through alternative translation start sites. Unlike p66^Shc^, the p52^Shc^ and p46^Shc^ proteins have been detected in almost all cell types^21^. The three protein isoforms of *Shc1* reportedly have distinct biological functions. *p66*^*Shc*^ knockout mice were protected against vascular, cardiac, and renal damage, while overexpression of *p66*^*Shc*^ led to cytochrome c release and apoptosis^22,23^. p52^Shc^, the most well studied isoform, acts as a bridge between growth factor receptors and the adaptor Grb2, which binds guanine nucleotide exchange factor Sos^24^ resulting in the activation of Ras and downstream MAPK signalling pathways. p46^Shc^ was recently reported to be a negative regulator of mitochondrial thiolase, which is involved in fatty acid metabolism^25^. Mice with low levels of p46^Shc^ are lean and resistant to obesity^25^. The expression levels for *Shc1* isoforms in our dataset, calculated by Salmon, are shown in Fig. 1. The comparison and statistical analysis results between obese and lean groups across different time points were summarized in Supplementary Table S2. The expression of the mRNA corresponding to *p66*^*Shc*^ (ENSRNOT00000028038) was elevated in obese animals at 12, 20, and 29 weeks but not at later time points, which may be linked to obesity-induced oxidative stress during the early and intermediate stages of DN. In contrast, the expression levels of the mRNA corresponding to *p46*^*Shc*^/*p52*^*Shc*^ (ENSRNOT00000056652) were similar between the lean and obese groups at 12, 20, 24, and 29 weeks of age, but reduced in obese animals at 34 and 41 weeks of age. This reduction may be indicative of dysregulated fatty acid metabolism and/or cell proliferation in late stage DN.

**Figure 2:**
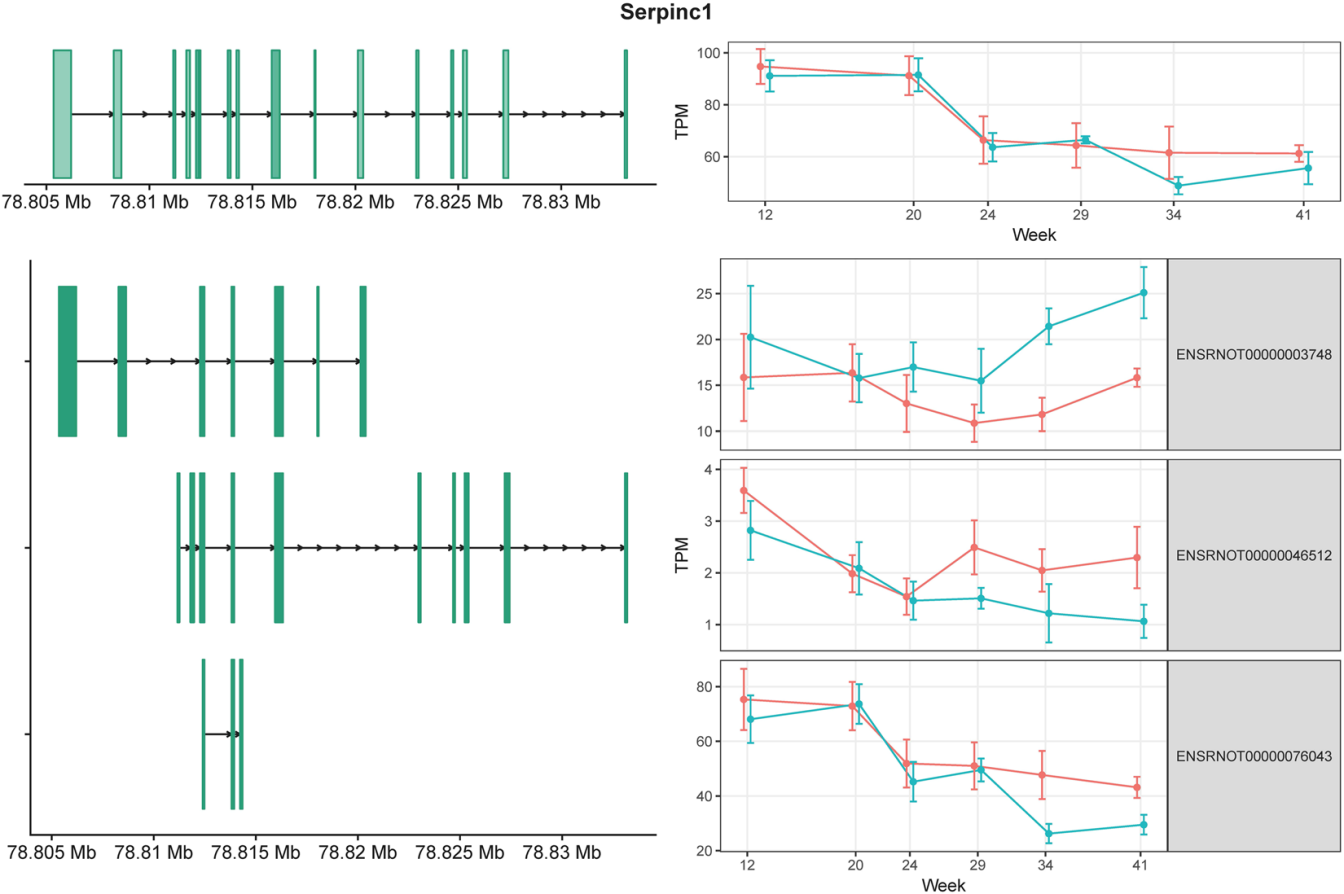
Exon structures and expression levels of *Serpinc1* and its three transcripts. *Serpinc1* is on the “+” strand. The exons are shown in green. The corresponding longitudinal TPM values estimated from the RNA-seq data are shown on the right. Lean and obese animals are indicated in red and blue, respectively. The error bars indicate the 95% confidence intervals.

*Serpinc1* (serpin family C member 1) encodes antithrombin III (ATIII), which is a serine protease inhibitor in the coagulation cascade^26^. Evidence suggests that ATIII deficiency is associated with kidney injury^26^. There are three annotated isoforms (Fig. 2), but their biological functions are unknown. In our analysis, ENSRNOT00000003748 showed higher expression in obese animals than in lean animals at weeks 34 and 41, whereas the expression levels of ENSRNOT00000046512 and ENSRNOT00000076043 at these time points were depressed in obese animals (Supplementary Table S2). Therefore, at these later time points, the direction of the expression changes was opposite for different isoforms, resulting in an expression profile that appeared relatively unchanged in the gene-level analysis (Fig. 2). This result illustrates how an isoform-level analysis of RNA-seq data can unmask more accurately changes in mRNA abundance.

**Figure 3:**
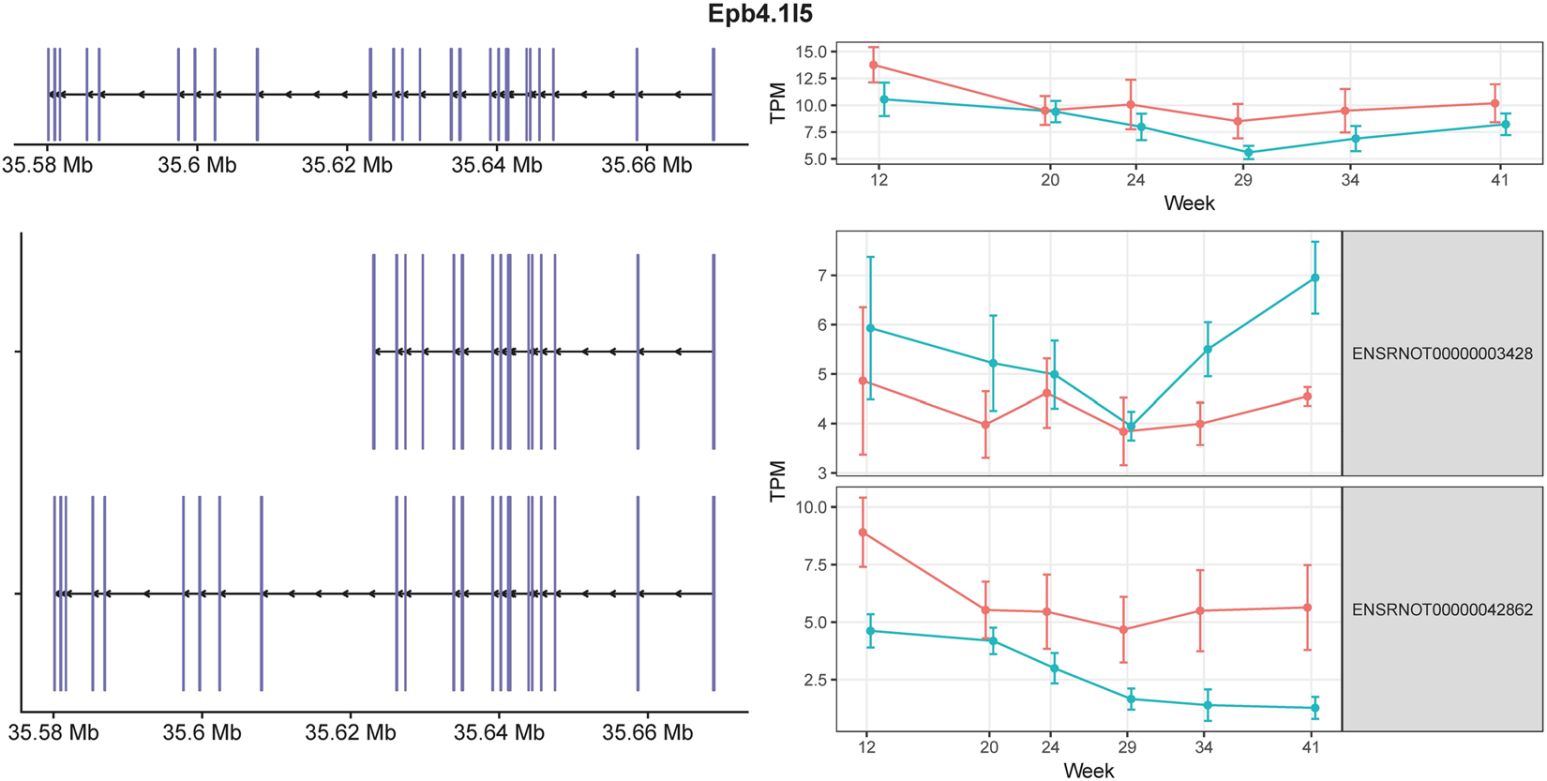
Exon structures and expression levels of *Epb4.1l5* and its two transcripts. *Epb4.1l5* is located on the “−” strand. The exons are shown in purple. The corresponding longitudinal TPM values estimated from the RNA-seq data are shown on the right. Lean and obese animals are indicated in red and blue, respectively. The error bars indicate the 95% confidence intervals.

*Epb4.1l5* (the erythrocyte protein band 4.1-like5) encodes a mesenchymal-specific protein that is induced during the process of epithelial-mesenchymal transition (EMT)^27^. Epb4.1l5 binds to p120 catenin, and causes E-cadherin internalization^28^. It also binds to paxillin, promotes focal adhesion dynamics, and has been implicated in cell motility^28^. The expression levels of the two annotated isoforms of *Epb4.1l5* are shown in Fig. 3. The comparison and statistical analysis results between obese and lean groups across different time points were summarized in Supplementary Table S2. ENSRNOT00000003428 had higher expression in the obese rats, while ENSRNOT00000042862 had higher expression in the lean controls. Although the biological functions of the isoforms of *Epb4.1l5* are not well studied, the consistent difference in isoform usage in obese and lean animals indicates the two isoforms may play different roles in EMT during DN progression^29^.

### RNA-seq analyses of known and novel splicing at the exon level

We next performed an exon-level analysis of the RNA-seq data to examine alternative splicing in an unbiased manner. Raw sequence reads were mapped to the Ensembl v87 annotated rat genome using STAR^30^. To find as many novel junctions as possible, the STAR 2-PASS protocol was used. The rMATS package was then applied to discover both annotated and novel exons, and to calculate differences in exon inclusion between obese and lean animals. The total numbers of sequencing reads were very close for all RNA samples (see Supplementary Table S1); therefore, the rMATS reported read counts were not further normalized. By way of illustration, below we describe our findings for *Il-33* that displayed alternative splicing patterns.

**Figure 4:**
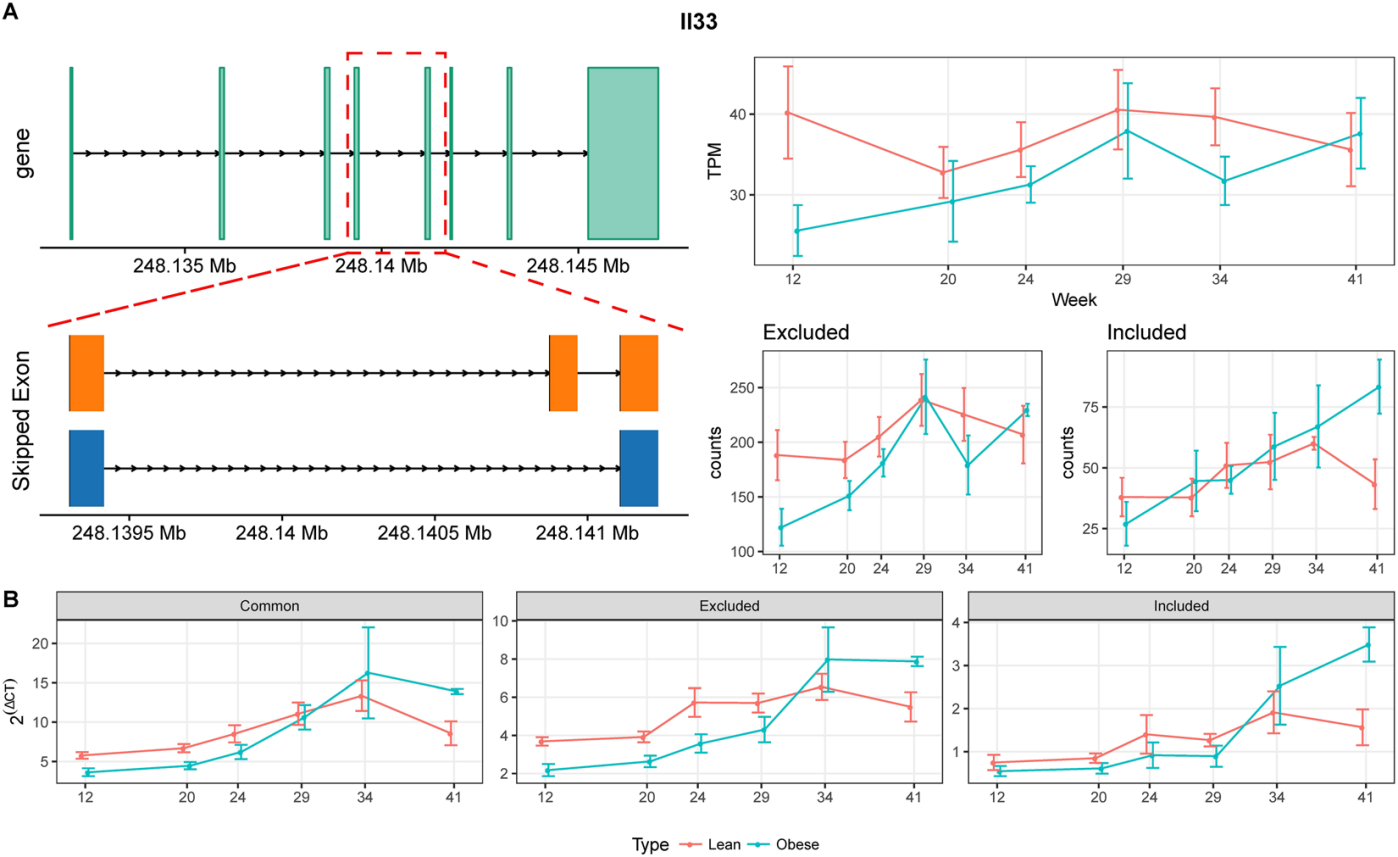
Differential splicing of *Il-33*. (**A**) Gene structure (top left) and the skipped exon event at the 3′ end of *Il-33* (bottom left). The gene-level TPM values and the corresponding read counts supporting exon inclusion and exclusion are shown on the right. (**B**) Expression values obtained by qRT-PCR. The expression values are 2^ΔCt^ normalized to the housekeeping gene Gusb. All the error bars indicate the 95% confidence intervals.

Interleukin (IL)-33 is a member of the IL-1 cytokine family, which is widely expressed in epithelial and endothelial cells, and which mediates tissue inflammation and repair responses^31–33^. Recent evidence from both clinical and preclinical studies indicates that Il-33 and its receptor ST2 (suppression of tumorigenicity 2) are involved in the pathogenesis of chronic kidney disease, systemic lupus nephritis, and acute kidney injury^34^. A novel skipped exon in *Il-33* was discovered in our analysis, and is illustrated in Fig. 4A. The exon-level RNA-seq analyses and qRT-PCR (Fig. 4B) both confirmed the presence of this novel exon. Moreover, the expression of the included isoform was higher in obese rats than in lean controls at week 41, whereas, for the excluded isoform, there was less of a difference between obese and lean animals at week 41 by both RNA-seq and qRT-PCR (Fig. 4). The comparison and statistical analysis results for gene expression, exon inclusion and exclusion between obese and lean groups across different time points were summarized in Supplementary Table S3. The qRT-PCR results matched well with the RNA-seq data in most cases, except for the excluded form in obese animals at week 34. Whether the included or excluded isoforms play different roles is not clear, and additional research is needed to uncover the function of this novel excluded isoform of *Il-33* identified from the exon-level RNA-seq data analysis.

**Figure 5:**
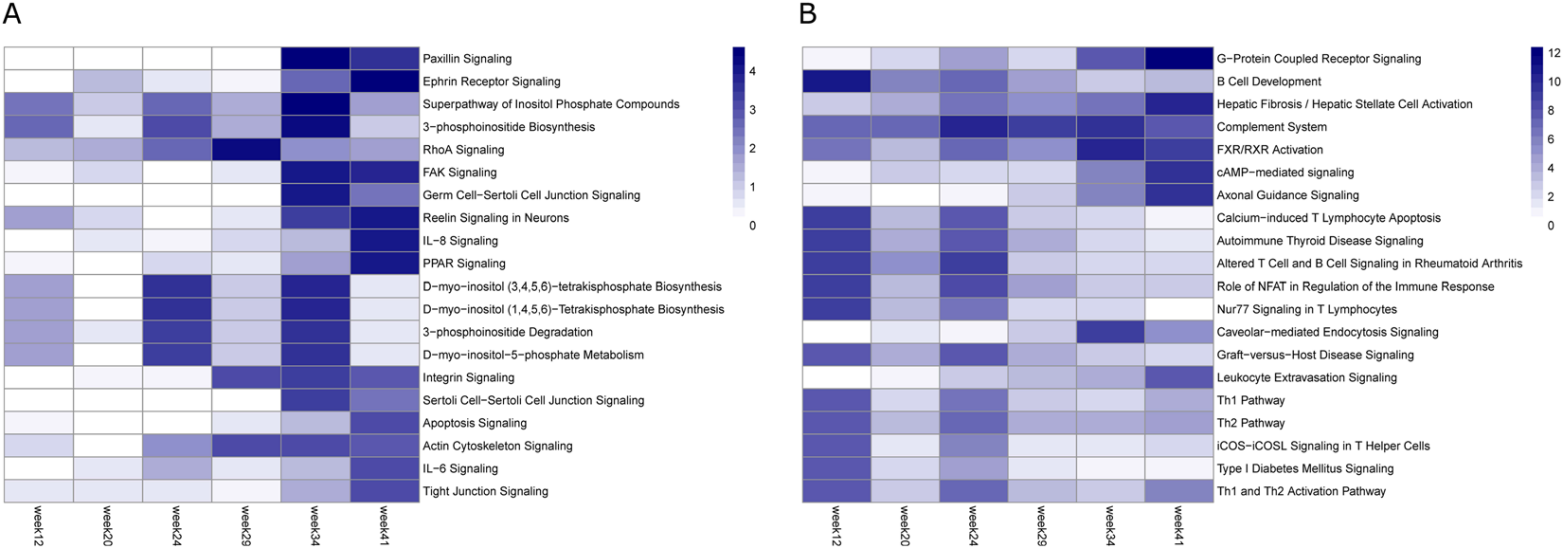
Top 20 enriched pathways associated with the differentially spliced genes (**A**) and differentially expressed genes (**B**). The top 20 canonical pathways were selected according to the smallest P values reported by IPA across all time points, and coloured based on the corresponding −log10 (P value).

After stringent filtering (details in methods section), the lists of genes with statistically significant differences at the exon inclusion level were uploaded to Ingenuity Pathway (IPA) for enrichment analysis. A total of 513 alternatively spliced genes mapped to the IPA database. The enriched canonical pathways in the exon-level analyses were ranked by the P values reported by IPA across all time points, and the top 20 pathways are shown in Fig. 5A. For comparison, the top 20 enriched pathways from a gene-level differential expression analysis, as opposed to a differentially spliced gene analysis, are shown in Fig. 5B. Different pathways were enriched between these two analyses. The alternatively spliced genes were enriched in pathways related to cell adhesion (paxillin signalling, FAK signalling, and integrin signalling), cell-cell interactions/junctions (ephrin receptor signalling, tight junction signalling, and germ cell–Sertoli cell junction signalling), cytoskeleton signalling, and inositol phosphate signalling, whereas the differentially expressed genes were enriched in pathways related to the immune response, G protein-coupled receptor, and cAMP signalling.

Podocytes are specialized epithelial cells that are essential to the glomerular filtration barrier^35^. Our previous study showed there were no significant changes in podocyte numbers per glomerulus between lean and obese animals, despite a significant decline in the abundance of podocyte-specific mRNAs^3^. This result, combined with observed podocyte foot process effacement by transmission electron microscopy, suggested that podocyte epithelial-mesenchymal transition (EMT) may be occurring in obese animals as they approached end-stage renal disease^36^. Consistent with this hypothesis, in the pathway analysis of genes showing differences in alternative splicing patterns, enriched pathways included cytoskeleton reorganization, changes in cell morphology, and mobility (Fig. 5A). Importantly, these pathways are heavily involved during the process of EMT^37,38^. There is also clinical and preclinical evidence that suggests a role for alternative splicing in cytoskeleton reorganization during EMT^39^. Therefore, the pathway enrichment of differentially spliced genes reveals potential additional mechanisms not revealed by the gene-level analyses. By combining both differential splicing and standard expression analyses, additional hypotheses with respect to mechanisms of disease pathogenesis can be formulated in this manner.

## Discussion

Recently, alignment-free tools, such as Kallisto^14^ and Salmon^15^, have been developed for transcript quantification. However, the accuracy of the quantification relies heavily on accurate isoform annotation, which is far from complete even for well-annotated organisms such as human and mouse. Exon-level analyses using tools such as MISO^17^, MAJIQ^18^, and rMATS^19^ can reveal novel isoforms and are especially attractive for less well-annotated species, such as rat.

Here we applied Salmon and rMATS to analyse RNA-seq data at the isoform- and exon-levels, respectively, in a rat model of type 2 DN. These analyses identified alternative splicing patterns in genes that may be implicated in disease pathogenesis (such as *Shc1*, *Serpinc1*, *Epb4.1l5*, and *Il-33*), which would have been overlooked in standard gene-level analysis. The novel splice form in *Il-*33 identified from our exon-level analysis was validated by qRT-PCR. In general, there was very good concordance between the RNA-seq data and the qRT-PCR analysis, although there were situations in which the results did not precisely align. We speculate there are three main reasons for the discrepancy. First, qRT-PCR can suffer from primer specificity and PCR artifacts^40^. Second, RNA-seq is vulnerable to the general biases and errors inherent in next-generation sequencing upon which it is based. The fragments are not uniformly sampled and sequenced due to preferential sites of fragmentation, and variable primer and tag nucleotide composition effects^41,42^. Third, the exon and isoform annotations may be incomplete or inaccurate. Our findings point to the necessity for orthogonal, experimental approaches to validate novel splicing events that are identified through bioinformatics approaches.

Alternative exons, especially minor-isofom ones, were shown to be less conserved among species than constitutive exons^43^. For a particular gene in rat, its alternative splicing pattern in different conditions is not necessarily the same as its splicing pattern in its orthologous gene in human. The results reported in this work are not translational. However, our point holds true that much more insights can be gained from alternative splicing analysis of RNA-seq data.

RNA-seq has become the *de facto* standard in transcriptome profiling, and most studies have focused only on gene-level analyses. However, mRNAs and proteins, rather than genes, are the actual functional units in living cells. In this study, we explored the role of alternative splicing during DN progression in glomeruli-enriched tissues of ZSF1 rats and demonstrated that broader and deeper biological insights can be obtained if RNA-seq data are analysed at the transcript and exon levels. Subtle differences, such as alternative isoform usage and exon inclusion/exclusion, can be missed by gene-level RNA-seq data analysis. Therefore, we believe that significant mechanistic insights can be gained from the simultaneous analysis of RNA-seq data at the gene, transcript, and exon levels.

## Materials and Methods

### RNA-seq isoform analyses

Raw sequencing reads were processed by Salmon^15^ 0.8.2 using Ensembl v87 rat annotation with default options. The resulting isoform-level counts tables were analysed using the R/Bioconductor package edgeR^20^ 3.16.5 for normalization and differential expression between lean and obese groups at each time point. The quasi-likelihood F-test glmQLFit was used instead of a likelihood ratio test because of the relatively small numbers of replicates at each time point.

### RNA-seq exon-level analyses

Raw sequencing reads were first mapped to the rat genome using STAR^30^ 2.5.2a with the 2-PASS option. The resulting 2-PASS bam files were processed by rMATS^19^ for exon inclusion level differences between lean and obese groups at each time point. Both exon–exon junction reads and the reads that mapped to the exons were used in the analyses. The resulting alternative splicing tables in five categories (alternative 5′ splice site, alternative 3′ splice site, mutually exclusive exons, retained introns, and skipped exons) were merged.

### Ingenuity pathway analyses

Pathway enrichment analyses were performed using IPA for both differentially expressed genes and differentially spliced genes. The Ensembl ID and FDR were used as input for pathway enrichment analyses. For the gene-level enrichment analyses, the following filters were applied: average read counts >2, log_2_fold_change >2, and FDR < 0.05. For the exon-level analyses, the filters were: FDR < 0.05, abs (inclusion level difference) >0.05, percent sample expressed >80%, and average read counts >10.

### qRT-PCR validation

Custom primer probe sequences labelled with 6-carboxyfluorescein (FAM) were ordered from Integrated DNA Technologies (IDT, Coralville, IA). The nucleotide sequences of these primers and probes, and their relative positions with respect to detection of alternative splice forms, are provided in Supplementary Table S4. The same total RNA samples that were subjected to RNA-seq were used for the qRT-PCR analysis. All qRT-PCR reactions were performed using a Taqman RNA-to-Ct 1-Step Kit (Thermo Fisher; catalogue number 4392656) following the manufacturer’s protocol, on an Applied Biosystems Quantstudio 12 K Flex Real-Time PCR System (Thermo Fisher). Ct values for each primer/probe set were normalized to the Ct values for the housekeeping gene *Gusb* for each sample to calculate ${2}^{{{\rm{\Delta }}}_{{\rm{ct}}}}$ values.

### Availability of data

The RNA-seq data in SRP094779 were downloaded from Sequence Read Archive.

## Electronic supplementary material

Supplementary Information
