# Supplementary material for: Computational identification and validation of alternative splicing in ZSF1 rat RNA-seq data, a preclinical model for type 2 diabetic nephropathy
Source: Sci Rep. 2018 May 16;8:7624. doi: 10.1038/s41598-018-26035-x (PMC5955895; doi:10.1038/s41598-018-26035-x)
Supplement: Supplementary file 1 — Supplementary Information [file 41598_2018_26035_MOESM1_ESM.pdf]

Computational identification and validation of alternative splicing in ZSF1 rat RNA-seq data, a preclinical model for type 2 diabetic nephropathy

Chi Zhang, Ken Dower, Baohong Zhang, Robert Martinez, Lih-Ling Lin and Shanrong Zhao

Table S1. Descriptions on RNA-seq samples and the library size of sequencing

Sample	Reads	Type	Week	Sample	Reads	Type	Week
**A1-PFE-ZSF1**	88112024	lean	12	D1-PFE-ZSF1	88136478	lean	29
**A2-PFE-ZSF1**	87994026	lean	12	D2-PFE-ZSF1	88543794	lean	29
**A3-PFE-ZSF1**	87920290	lean	12	D3-PFE-ZSF1	88013002	lean	29
**A4-PFE-ZSF1**	88143212	lean	12	D4-PFE-ZSF1	87497798	lean	29
**A5-PFE-ZSF1**	87606542	lean	12	D5-PFE-ZSF1	88744782	lean	29
**A6-PFE-ZSF1**	86325474	obese	12	D6-PFE-ZSF1	88895756	obese	29
**A7-PFE-ZSF1**	86286378	obese	12	D7-PFE-ZSF1	89176152	obese	29
**A8-PFE-ZSF1**	88134058	obese	12	D8-PFE-ZSF1	89286744	obese	29
**A9-PFE-ZSF1**	90169140	obese	12	D9-PFE-ZSF1	89591378	obese	29
**A10-PFE-ZSF1**	88116962	obese	12	D10-PFE-ZSF1	89392328	obese	29
**B1-PFE-ZSF1**	87998376	lean	20	E1-PFE-ZSF1	89389008	lean	34
**B2-PFE-ZSF1**	88106294	lean	20	E2-PFE-ZSF1	89317832	lean	34
**B3-PFE-ZSF1**	89640408	lean	20	E3-PFE-ZSF1	89375118	lean	34
**B4-PFE-ZSF1**	89593368	lean	20	E4-PFE-ZSF1	86947992	lean	34
**B5-PFE-ZSF1**	89335280	lean	20	E5-PFE-ZSF1	89474602	lean	34
**B6-PFE-ZSF1**	87611102	obese	20	E6-PFE-ZSF1	87178270	obese	34
**B7-PFE-ZSF1**	87554564	obese	20	E7-PFE-ZSF1	88859810	obese	34
**B8-PFE-ZSF1**	87382592	obese	20	E8-PFE-ZSF1	85905608	obese	34
**B9-PFE-ZSF1**	87430520	obese	20	E9-PFE-ZSF1	87504152	obese	34
**B10-PFE-ZSF1**	87575600	obese	20	E10-PFE-ZSF1	85926790	obese	34
**C1-PFE-ZSF1**	89504534	lean	24	F1-PFE-ZSF1	88440452	lean	41
**C2-PFE-ZSF1**	87055654	lean	24	F2PFE-ZSF1	87786394	lean	41
**C3-PFE-ZSF1**	87918752	lean	24	F3-PFE-ZSF1	87663194	lean	41
**C4-PFE-ZSF1**	88108878	lean	24	F4-PFE-ZSF1	87558780	lean	41
**C5-PFE-ZSF1**	89914574	lean	24	F6-PFE-ZSF1	87731544	obese	41
**C6-PFE-ZSF1**	87804330	obese	24	F7-PFE-ZSF1	87783398	obese	41
**C7-PFE-ZSF1**	90019774	obese	24	F8-PFE-ZSF1	89252478	obese	41
**C8-PFE-ZSF1**	88358488	obese	24				
**C9-PFE-ZSF1**	87779510	obese	24				
**C10-PFE-ZSF1**	88641626	obese	24				

Table S2. Statistical analysis (for genes Shc1, Serpinc1,and Epb4.1l5) between obese and lean groups across time points. Note the unit for difference is TPM; and only those time points with statistical significant are listed.

Gene_Name	Week	Entity	ID	Difference	P_Value
Shc1	12	Gene	ENSRNOG00000020657	-2.33	5.40E-01
Shc1	12	Isoform	ENSRNOT00000028038	-3.22	1.00E-02
Shc1	12	Isoform	ENSRNOT00000056652	0.89	7.48E-01
Shc1	20	Gene	ENSRNOG00000020657	-3.11	1.90E-01
Shc1	20	Isoform	ENSRNOT00000028038	-2.35	4.60E-03
Shc1	20	Isoform	ENSRNOT00000056652	-0.75	6.48E-01
Shc1	29	Gene	ENSRNOG00000020657	-1.90	3.65E-01
Shc1	29	Isoform	ENSRNOT00000028038	-3.08	3.47E-05
Shc1	29	Isoform	ENSRNOT00000056652	1.18	4.72E-01
Shc1	34	Gene	ENSRNOG00000020657	11.65	7.83E-06
Shc1	34	Isoform	ENSRNOT00000028038	1.10	9.24E-03
Shc1	34	Isoform	ENSRNOT00000056652	10.55	3.26E-06
Shc1	41	Gene	ENSRNOG00000020657	5.64	9.59E-04
Shc1	41	Isoform	ENSRNOT00000028038	0.60	1.09E-01
Shc1	41	Isoform	ENSRNOT00000056652	5.05	6.31E-04
Serpinc1	34	Gene	ENSRNOG00000002783	12.66	6.83E-02
Serpinc1	34	Isoform	ENSRNOT00000003748	-9.61	1.09E-04
Serpinc1	34	Isoform	ENSRNOT00000046512	0.83	5.10E-02
Serpinc1	34	Isoform	ENSRNOT00000076043	21.45	6.03E-03
Serpinc1	41	Gene	ENSRNOG00000002783	5.62	2.11E-01
Serpinc1	41	Isoform	ENSRNOT00000003748	-9.27	1.42E-02
Serpinc1	41	Isoform	ENSRNOT00000046512	1.23	1.94E-02
Serpinc1	41	Isoform	ENSRNOT00000076043	13.66	4.03E-03
Epb4.1l5	12	Gene	ENSRNOG00000002538	3.21	2.38E-02
Epb4.1l5	12	Isoform	ENSRNOT00000003428	-1.07	3.43E-01
Epb4.1l5	12	Isoform	ENSRNOT00000042862	4.28	2.66E-03
Epb4.1l5	20	Gene	ENSRNOG00000002538	0.10	9.05E-01
Epb4.1l5	20	Isoform	ENSRNOT00000003428	-1.24	7.66E-02
Epb4.1l5	20	Isoform	ENSRNOT00000042862	1.34	1.04E-01
Epb4.1l5	34	Gene	ENSRNOG00000002538	2.60	7.01E-02
Epb4.1l5	34	Isoform	ENSRNOT00000003428	-1.51	3.22E-03
Epb4.1l5	34	Isoform	ENSRNOT00000042862	4.10	7.50E-03
Epb4.1l5	41	Gene	ENSRNOG00000002538	1.96	1.24E-01
Epb4.1l5	41	Isoform	ENSRNOT00000003428	-2.41	1.79E-02
Epb4.1l5	41	Isoform	ENSRNOT00000042862	4.37	1.58E-02

Table S3. Statistical analysis for gene expression, exon inclusion and exclusion between obese and lean groups across time points. Note: the unit for gene expression difference is TPM; whereas the unit for inclusion/exclusion difference are raw read count.

Gene_Name	Week	Entity	ID	Difference	P_Value
Il33	12	Gene	ENSRNOG00000016456	14.63	4.24E-03
Il33	12	Exon	excluded	66.00	2.31E-03
Il33	12	Exon	included	11.00	1.10E-01
Il33	20	Gene	ENSRNOG00000016456	3.56	2.77E-01
Il33	20	Exon	excluded	32.60	1.78E-02
Il33	20	Exon	included	-6.80	3.94E-01
Il33	24	Gene	ENSRNOG00000016456	4.32	7.71E-02
Il33	24	Exon	excluded	23.80	7.12E-02
Il33	24	Exon	included	6.00	3.17E-01
Il33	29	Gene	ENSRNOG00000016456	2.63	5.22E-01
Il33	29	Exon	excluded	-2.80	8.98E-01
Il33	29	Exon	included	-6.40	4.99E-01
Il33	34	Gene	ENSRNOG00000016456	7.93	1.04E-02
Il33	34	Exon	excluded	46.20	3.74E-02
Il33	34	Exon	included	-7.00	4.64E-01
Il33	41	Gene	ENSRNOG00000016456	-2.03	5.57E-01
Il33	41	Exon	excluded	-22.67	1.90E-01
Il33	41	Exon	included	-40.08	4.35E-03

Table S4. A list of primer sequences for qRT-PCR validation

Il33 unannotated

Inc (sense probe):

GTAGCAAGCATGAAGGGAGGCTCTCTACCTGTTTACCAGATTCTAGGAAGAGATCCCTGCTTGGCAGTATCCAAGCGTTTGCTGCATCAGTTGACACACTGAGTATCCAAGattgtaagaaggtaaagaagggaactgtcttcctcgttcctacccc**ccactgtgcaggaagctaacacct**ttaatcccaccctgggatcagGAACTTCACT**GCTAACAGAATCTTGTGCCCTGAG**CACATACAACGACCAATCTGTTAGTTTTGTTTTGGAGAATGGATGTTACGTGATCAATGTTGAAGATTGTGGGAAAAACCAAGAAAAAG

Ex (forward probe):

GTAGCAAGCATGAAGGGAGGCTCTCTACCTGTTTACCAGATTCTAGGAAGAGATCCCTGCTTGGCAGTATCCAAGCGTTTGCTGCATCAGTTGACACACTGAGTATCCAA**GG**AACTTCACTGCTAACAGAATCTTGTGCCCTGAGCACATACAACGACCAATCTGTTAGTTTTGTTTTGGAGAATGGATGTTACGTGATCAATGTTGAAGATTGTGGGAAAAACCAAGAAAAAG

Com (forward probe):

TAGCAAGCATGAAGGGAGGCTCTCTACCTGTTTACCAGATTCTAGGAAGAGATCCCTGCTTGGCAGTATCCAAGCGTTTGCTGCATCAGTTGACACACTGAGTATCCAA**GG**AACTTCACTGCTAACAGAATCTTGTGCCCTGAGCACATACAACGACCAATCTGTTAGTTTTGTTTTGGAGAATGGATGTTACGTGATCAATGTTGAAGATTGTGGGAAAAACCAAGAAAAAG

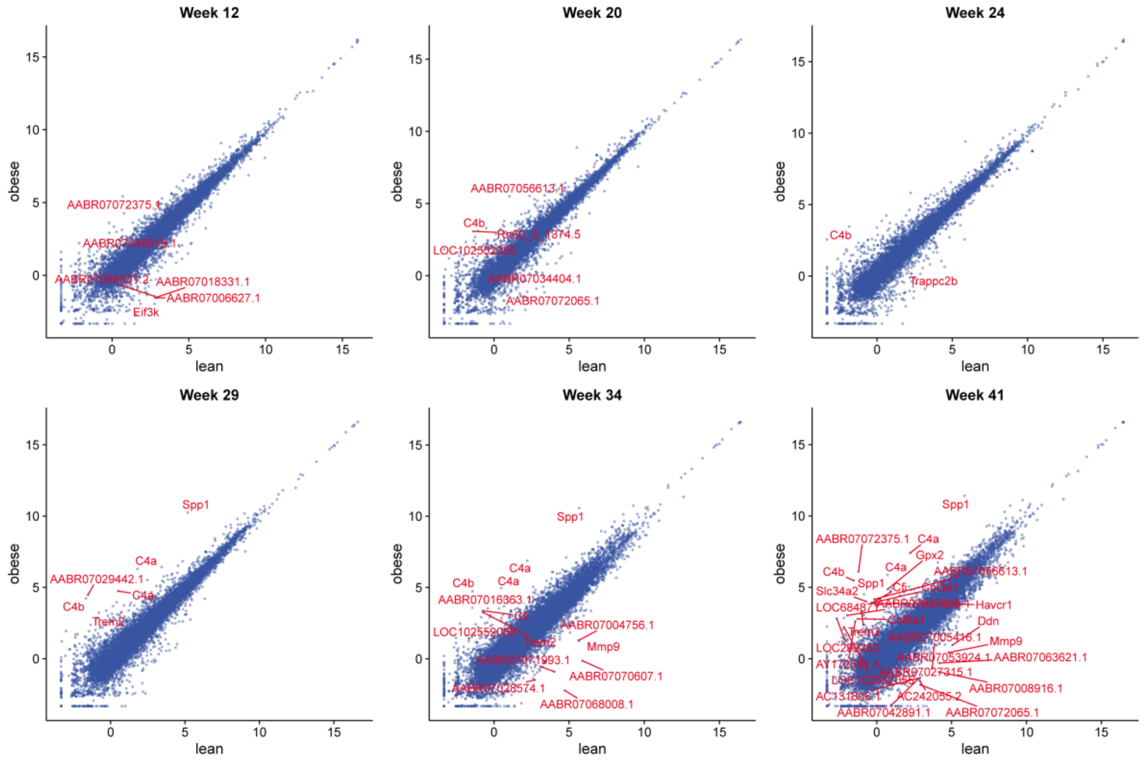

Figure S1. Scatter plots of obese versus lean transcript expression at each time point. The group average $\log_2$TPM values of obese and lean animals are plotted against one another at each time point. Transcripts with over 16 fold TPM difference are labelled.
